# Epstein-Barr Virus-Positive Nonkeratinizing Squamous Cell Carcinoma (Lymphoepithelioma-Like Carcinoma) of the Medial Periorbital Area: A Case Report

**DOI:** 10.7759/cureus.115740

**Published:** 2026-09-03

**Authors:** Stella Hayrapetyan, Isabel Randolph, Nithin Thummala, Fulvio Lonardo

**Affiliations:** 1 Pathology, Detroit Medical Center, Detroit, USA; 2 Otolaryngology, Detroit Medical Center, Detroit, USA; 3 Radiology, The Barbara Ann Karmanos Cancer Institute, Detroit, USA; 4 Pathology, The Barbara Ann Karmanos Cancer Institute, Detroit, USA

**Keywords:** eber, ebv, head and neck malignancies, lymphoepithelioma-like carcinoma, medial canthus mass, nonkeratinizing carcinoma, orbital ct imaging, orbital exenteration, periorbital tumor, squamous cell carcinoma

## Abstract

Epstein-Barr virus (EBV)-positive nonkeratinizing squamous cell carcinoma (NKSCC), formerly referred to as lymphoepithelioma or lymphoepithelioma-like carcinoma, most commonly arises in the nasopharynx. However, it has been reported in multiple extra-nasopharyngeal sites. Orbital involvement is exceedingly rare, with most reported cases occurring in the ocular adnexa. We present the case of a 67-year-old Asian male with EBV-positive NKSCC of the medial periorbital area who developed right-sided diplopia and periocular edema. Recognition of this rare entity and its diagnostic features is essential for accurate diagnosis and appropriate management.

## Introduction

Epstein-Barr virus-positive nonkeratinizing squamous cell carcinoma (EBV-NKSCC) is a rare, high-grade carcinoma characterized by distinctive morphologic features, including nests of pleomorphic epithelial cells with nucleomegaly and prominent nucleoli, arranged either in small clusters or in large syncytial nests, associated with a dense lymphoplasmacytic infiltration. This histologic pattern has historically been known as lymphoepithelioma (LE) or lymphoepithelioma-like carcinoma (LELC) [1].

Although most commonly arising in the nasopharynx, tumors with such morphology have been described in multiple extra-nasopharyngeal sites, including the lung and major salivary glands [2]. Sporadic cases have also been reported in other anatomic sites, such as the larynx, temporal bone, and middle ear [3].

Tumors involving the orbit may be primary or secondary, and their histological types differ based on precise anatomical localization and the patient’s age [4]. LELC has rarely been reported to arise in the lacrimal glands [5,6]. Our case enriches this limited series and demonstrates its association with EBV infection, cementing its similarity with nasopharyngeal tumors. It also firmly places this entity among the differential diagnostic considerations of orbital mass lesions, particularly those that pose a challenge to pathologists for the presence of a prominent lymphocytic component, such as orbital lymphoma and orbital inflammatory pseudotumors [4].

## Case presentation

A 67-year-old man of Asian ancestry with no significant past medical history presented with several months of progressive right orbital swelling, diplopia, and impaired adduction of the right eye. Physical examination revealed marked right periorbital edema without impairment of visual acuity, as well as prominent right cervical lymphadenopathy. The patient denied constitutional symptoms, including fever, weight loss, and night sweats.

Computed tomography (CT) of the orbits and neck with intravenous contrast demonstrated a 3.1-cm enhancing soft tissue mass centered at the right medial canthus. The lesion exerted mass effect on the right medial rectus muscle with minimal lateral and anterior displacement of the right globe, extended into and occluded the nasolacrimal duct, and demonstrated subtle osseous erosion of the adjacent anterior right ethmoid air cells (Figure 1). Bulky right cervical lymphadenopathy was also present, with the largest lymph node measuring up to 3.2 cm in the right level II nodal station, suspicious for metastatic disease (Figure 2). No additional abnormalities were identified elsewhere in the head and neck to suggest a separate nasopharyngeal or sinonasal primary. The tumor was centered in the medial periorbital region, with associated cervical lymph-node metastases. However, given involvement of the nasolacrimal duct, an occult primary arising from the lacrimal drainage apparatus could not be completely excluded.

**Figure 1:**
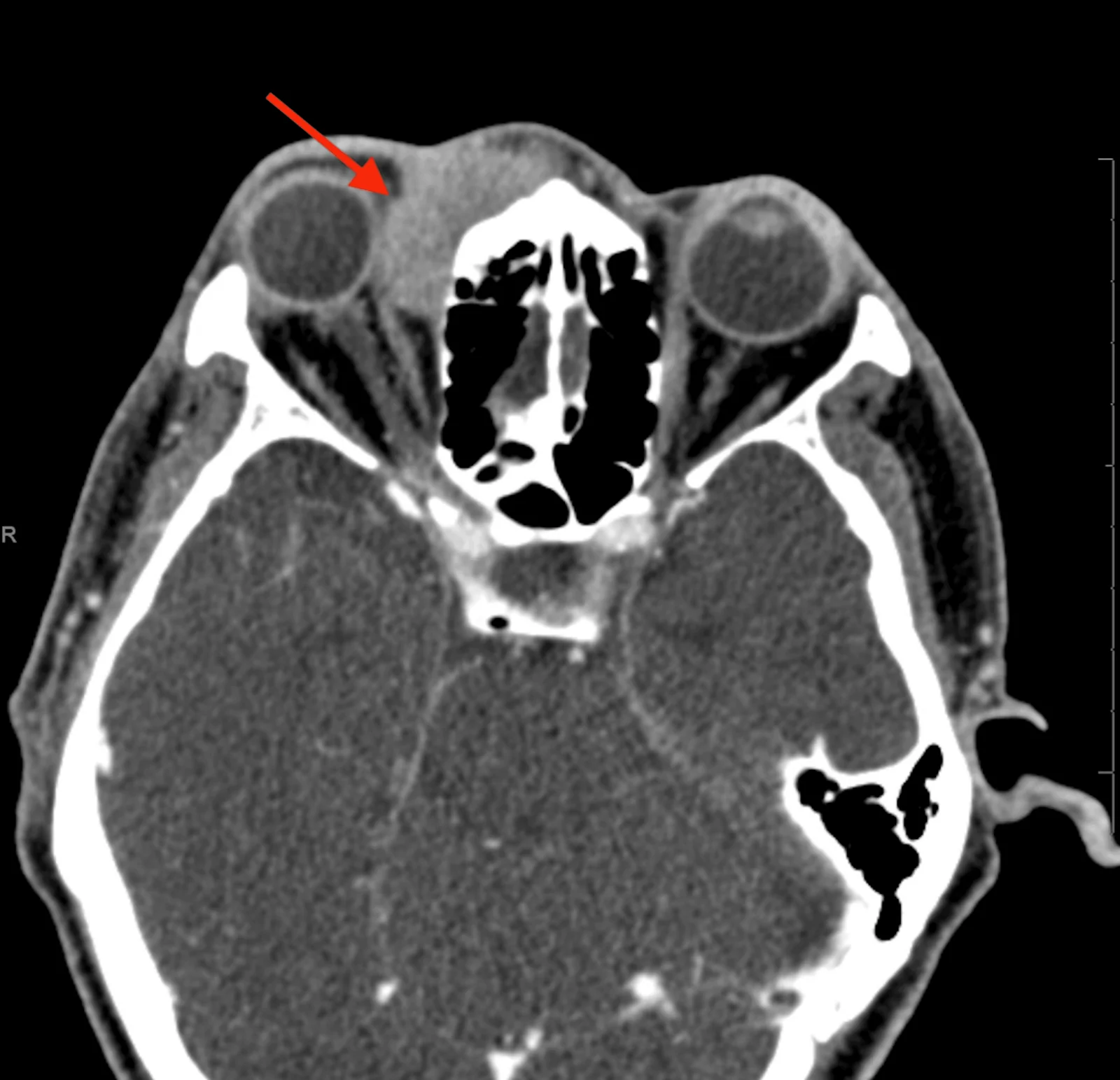
Contrast-enhanced computed tomography (CT) of the orbits. Axial view illustrating a soft tissue mass (red arrow) centered at the medial canthus of the right orbit, causing a mass effect on the right medial rectus muscle with minimal lateral and anterior displacement of the right globe.

**Figure 2:**
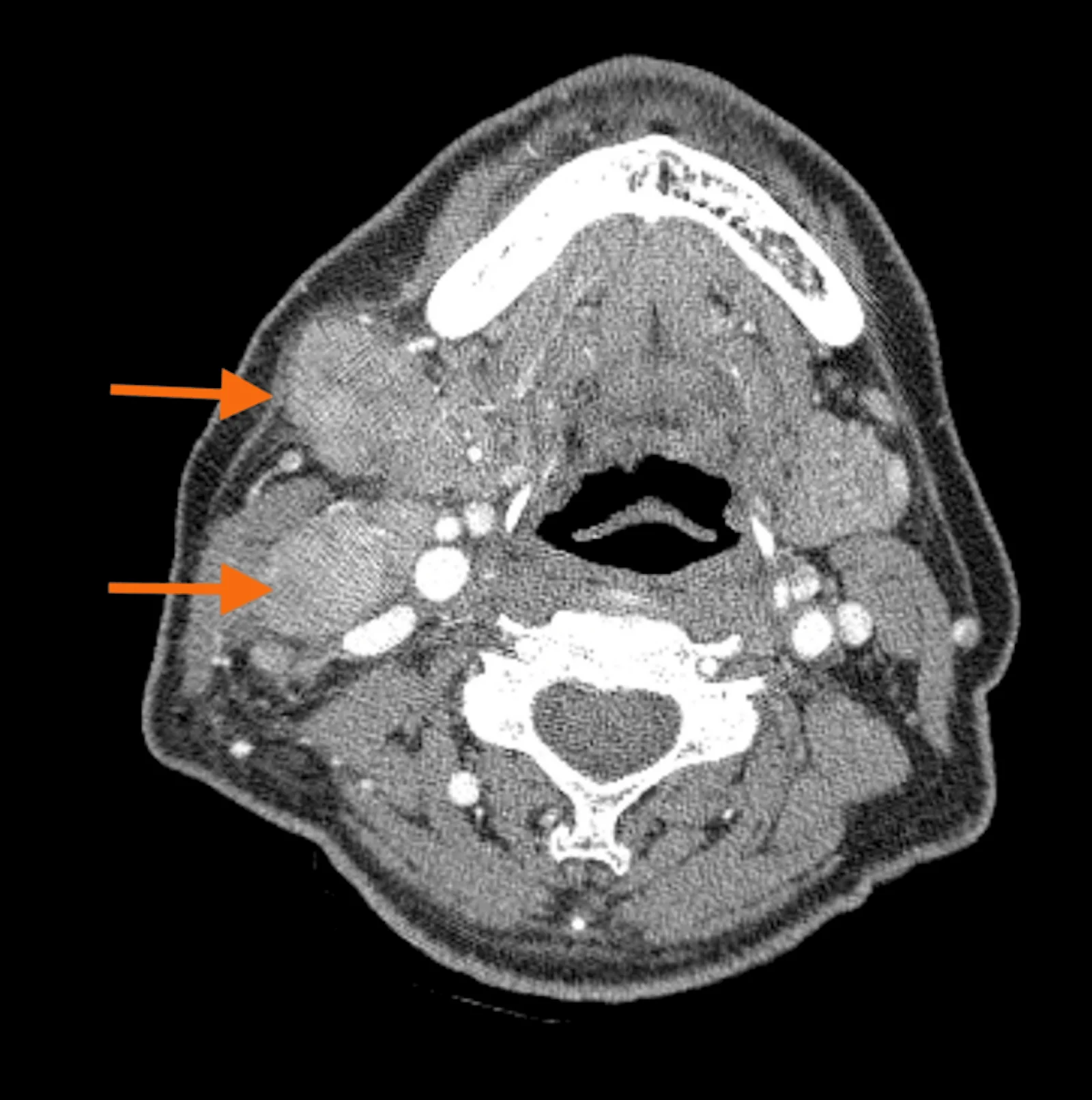
Contrast-enhanced computed tomography (CT) of the neck. Axial view showing bulky right cervical lymphadenopathy (orange arrows) involving the upper cervical nodal stations.

Ultrasound-guided core needle biopsy of the right cervical lymph node demonstrated diffuse and small clusters of large tumor cells with high nuclear-to-cytoplasmic ratios, vesicular chromatin, and prominent nucleoli, admixed with lymphocytes (Figure 3). Tumor cells were positive for broad-spectrum cytokeratin and p40, and negative for CK5/6, p16, synaptophysin, chromogranin, and neurofilament. Epstein-Barr virus in situ hybridization (EBV-ISH) demonstrated focal positivity (Table 1). Therefore, the tumor was classified as metastatic undifferentiated carcinoma.

**Figure 3:**
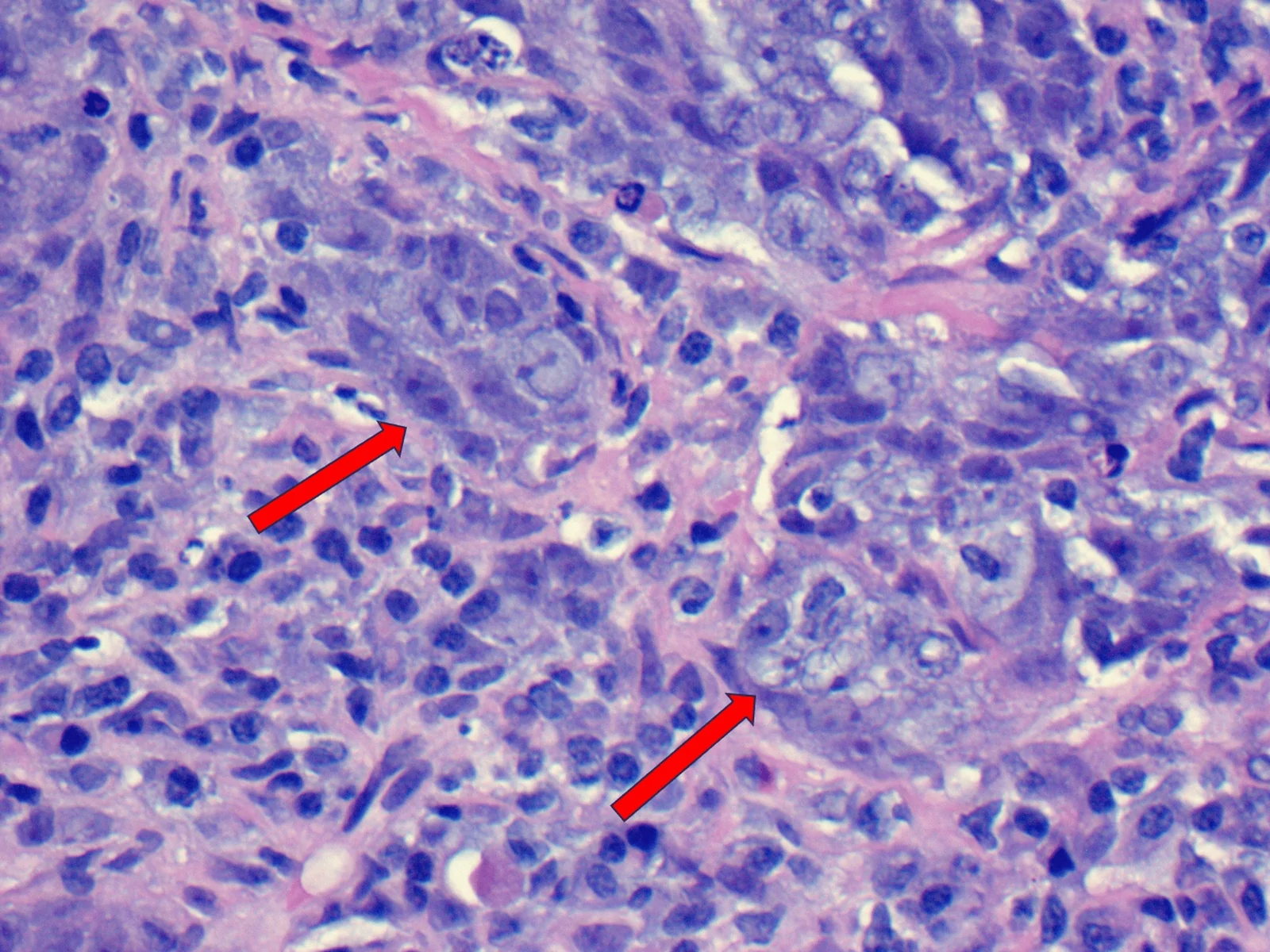
High-power (400×) histopathologic view of the specimen, hematoxylin & eosin (H&E) staining. Red arrows point to diffuse and small clusters of large tumor cells with high nuclear-to-cytoplasmic ratios, vesicular chromatin, and prominent nucleoli.

**Table 1:** Immunohistochemical and Epstein-Barr virus (EBV) in situ hybridization profile of the tumor.

Marker	Result	Interpretation
P40	Positive	Squamous differentiation
CAM 5.2	Positive	Epithelial origin
EBER ISH	Positive	EBV association

The patient then underwent surgical resection, which included right orbital exenteration, partial rhinectomy, maxillectomy, external ethmoidectomy, right parotidectomy, and right modified radical neck dissection. The resulting surgical defect was reconstructed using a free flap from the anterolateral thigh.

Gross examination of the resected specimen revealed a pale gray-pink, solid, irregularly contoured, rubbery mass measuring 3.9 × 3.0 × 2.8 cm. The surgical specimens were received fresh, routinely processed, and submitted for histologic evaluation.

Microscopic examination of the tumor demonstrated fibrous septa delineating ill-defined nodules composed of malignant epithelial cells with high nuclear‑to‑cytoplasmic ratios, vesicular chromatin, and prominent nucleoli. The tumor cells were arranged in small syncytial-like nests accompanied by a dense lymphoplasmacytic infiltrate (Figures 4-6), consistent with a classic lymphoepithelioma‑like morphology.

**Figure 4:**
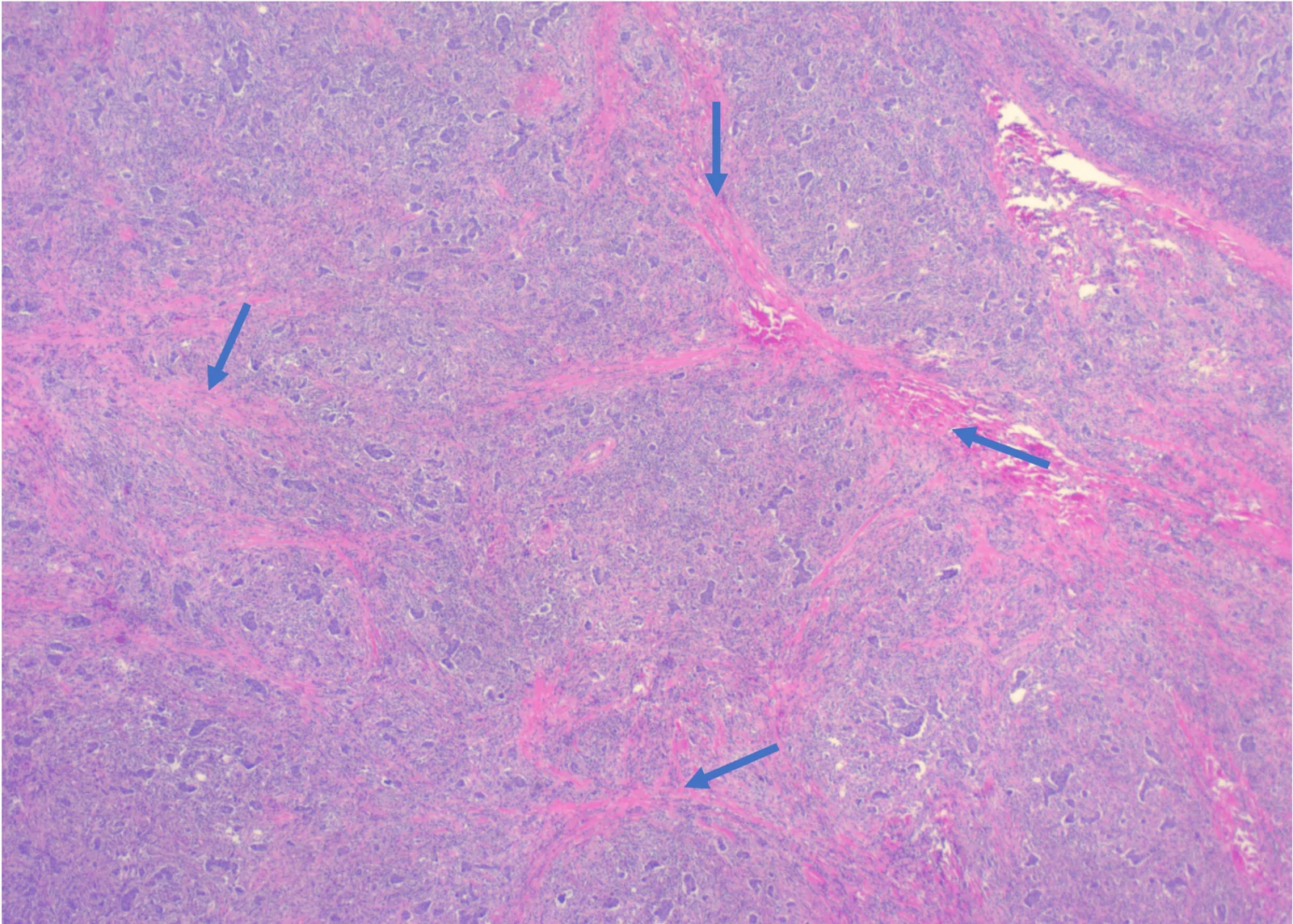
Low-power (20×) view of the tumor, hematoxylin & eosin (H&E) staining. Blue arrows point to fibrous septa partitioning the tumor into ill-defined nodules.

**Figure 5:**
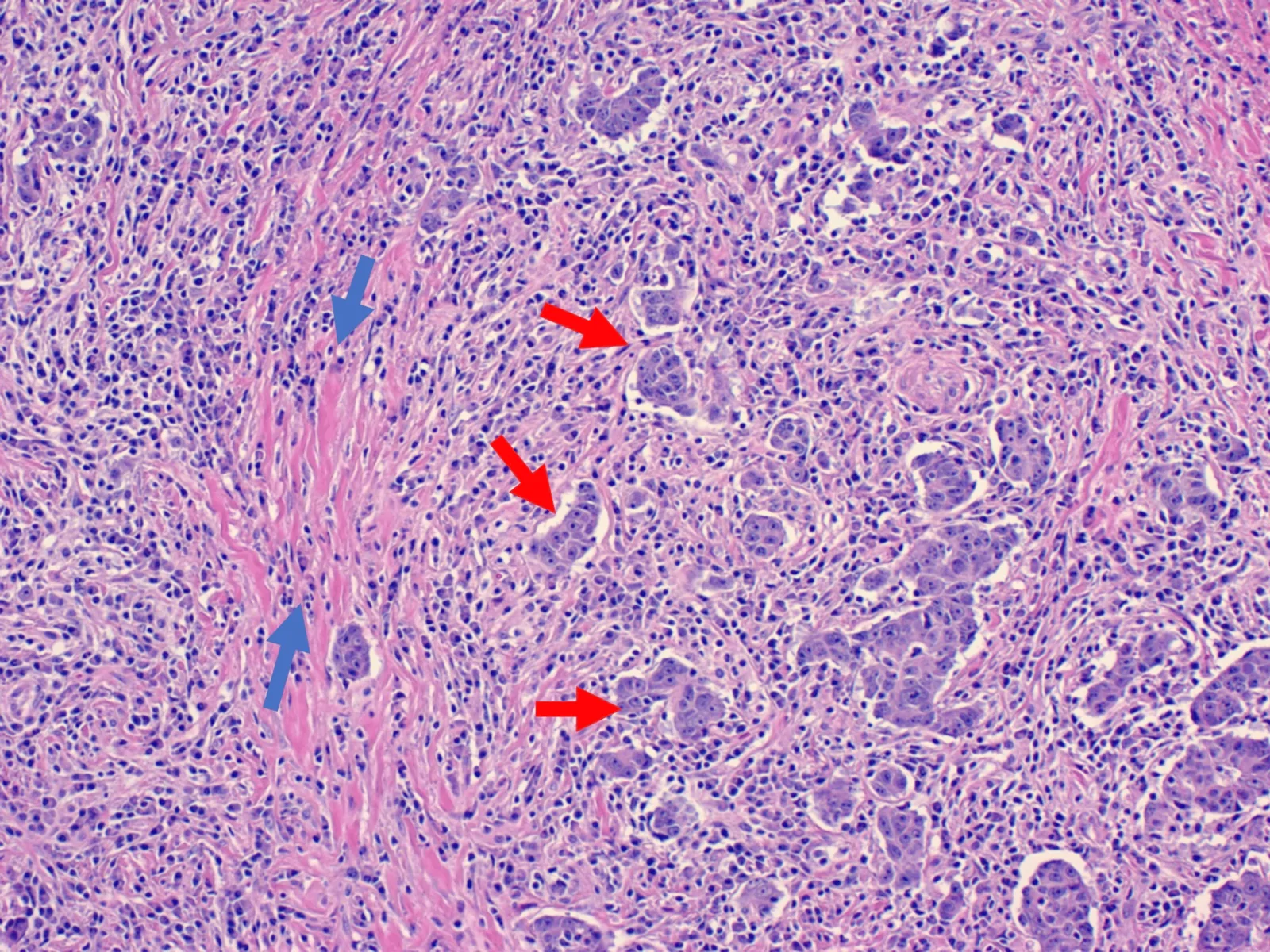
Intermediate-power (100×) view of the tumor, hematoxylin & eosin (H&E) staining. Blue arrows point to fibrous septa that delimit a tumor nodule, containing nests of epithelial cells (red arrows) interspersed with a dense lymphoplasmacytic infiltrate (small round blue cells).

**Figure 6:**
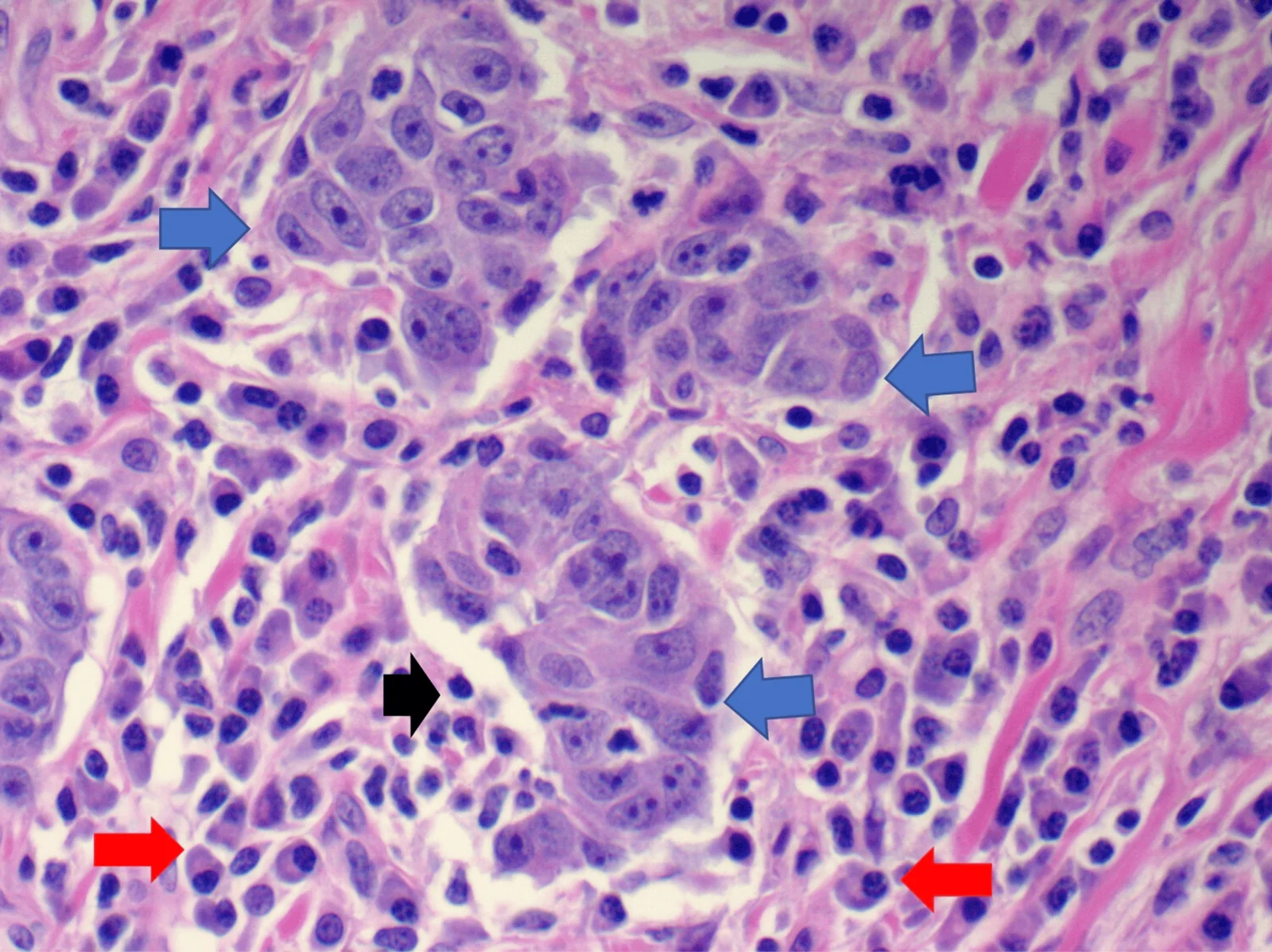
High-power (400×) view of the tumor, hematoxylin & eosin (H&E) staining. Demonstrating large epithelioid tumor cells with prominent nucleoli and indistinct cell borders arranged in a syncytial growth pattern (blue arrows), surrounded by lymphocytes (black arrow) and plasma cells (red arrows).

Immunohistochemical studies showed strong cytoplasmic positivity of the epithelial cells for CAM5.2, confirming their epithelial origin, and strong nuclear positivity for p40, supporting their squamous differentiation (Figures 7, 8). In situ hybridization for Epstein-Barr virus‑encoded RNA (EBER ISH) demonstrated diffuse nuclear positivity within the tumor cells, establishing EBV association (Figure 9).

**Figure 7:**
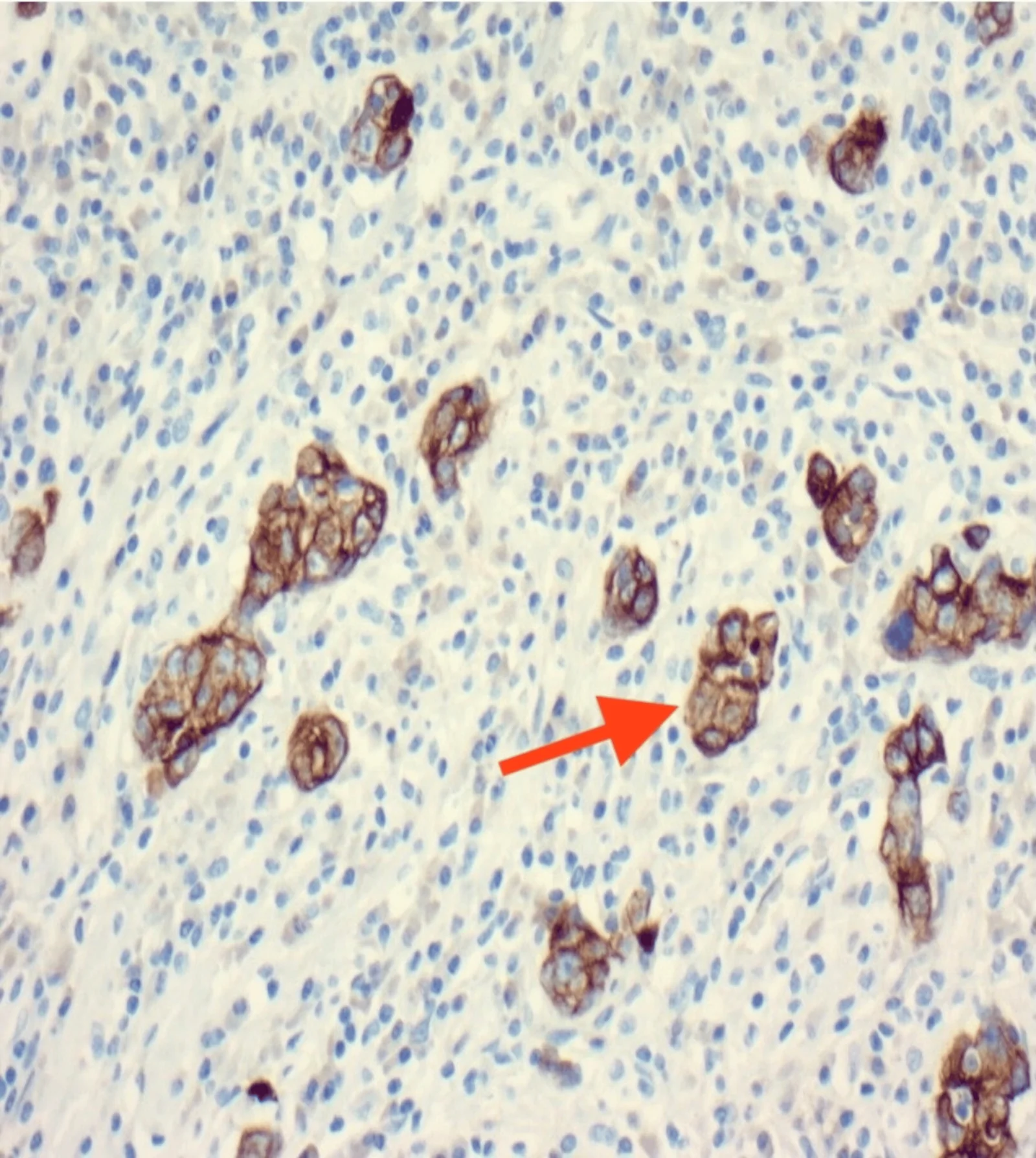
CAM 5.2 immunohistochemical staining of the tumor. Demonstrating cytoplasmic positivity (orange arrow) in the tumor cells.

**Figure 8:**
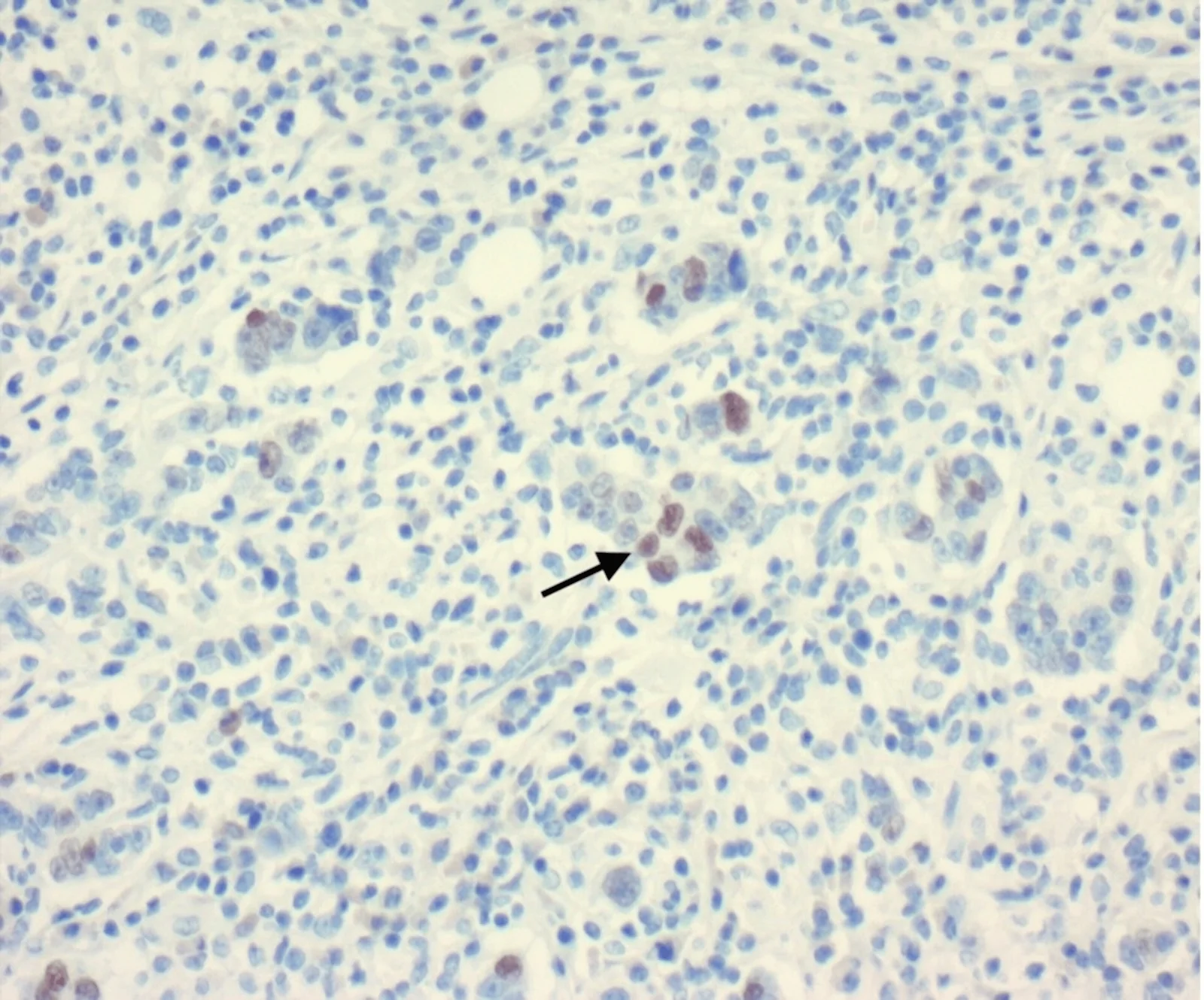
P40 Immunohistochemical staining of the tumor. Demonstrating patchy nuclear positivity (black arrow) in the tumor cells.

**Figure 9:**
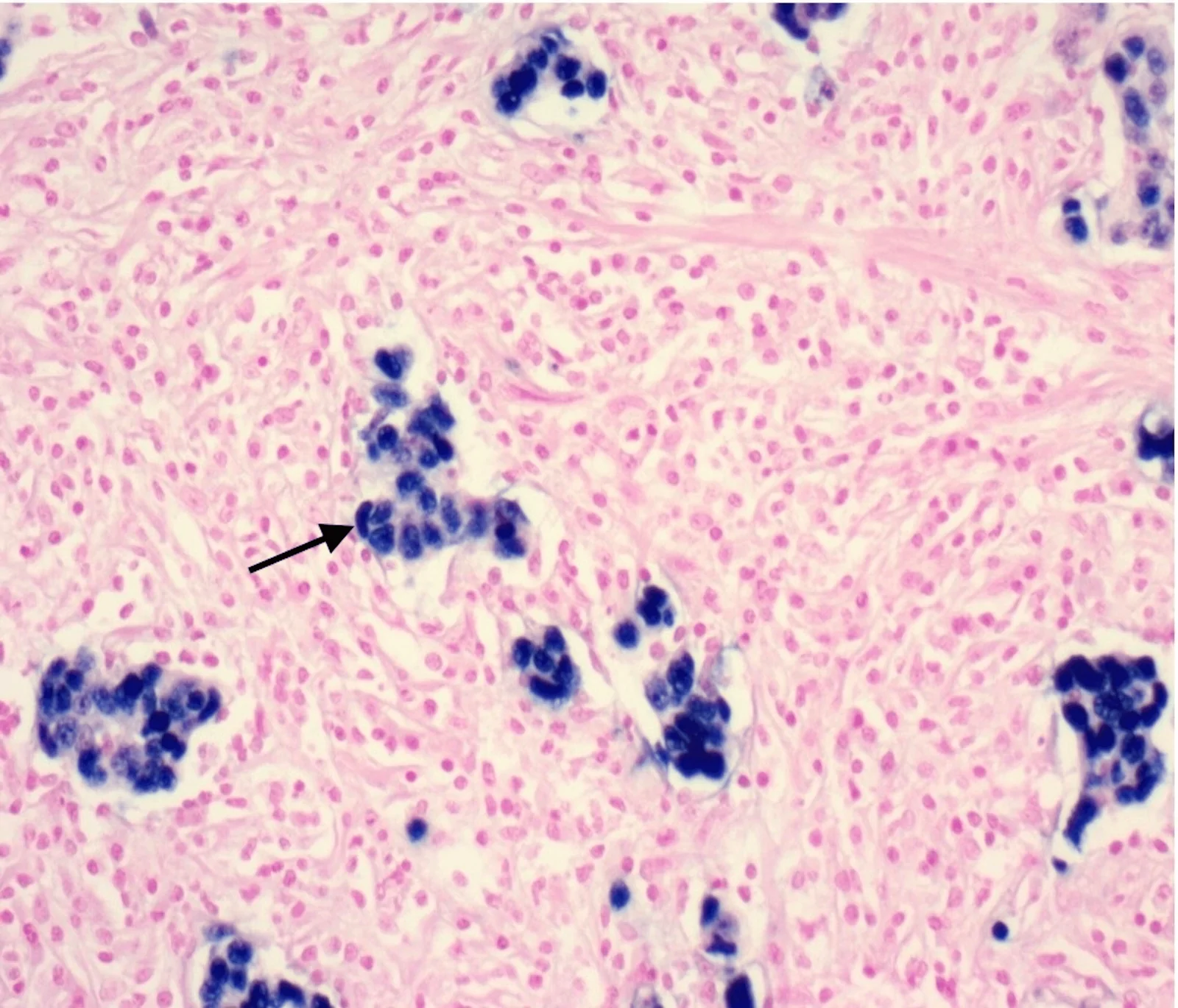
Epstein-Barr virus-encoded small RNA (EBER) in situ hybridization of the tumor. Demonstrating nuclear positivity in the tumor cells (black arrow).

Together, the morphologic appearance, immunophenotypic profile, and EBV positivity supported the diagnosis of EBV-NKSCC. Metastatic tumor was identified in cervical lymph node levels I through IV, involving 56 of 57 examined lymph nodes.

## Discussion

Primary epithelial malignancies involving the orbit are distinctly uncommon compared with lymphoid, inflammatory, vascular, and metastatic lesions [4]. Yet, NKSCC represents an exceptionally rare entity in these anatomical locations. In a series of 1,422 orbital tumors, only a single case of metastatic lymphoepithelioma was identified, underscoring the rarity of NKSCC in the orbital region [7].

Nasopharyngeal carcinoma most commonly exhibits NKSCC histology, particularly in endemic regions such as Southeast Asia, where the non-keratinizing subtype is strongly associated with EBV infection [8]. Tumors with similar lymphoepithelioma-like morphology have also been described in multiple extra-nasopharyngeal sites. Orbital and ocular adnexal involvement remains exceptionally uncommon, and EBV positivity has been inconsistently demonstrated in previously reported cases, several of which were EBV-negative [5,6,9]. However, EBV-positive orbital cases have also been reported [10,11].

Most reported orbital NKSCCs with lymphoepithelioma-like morphology appear to arise from ocular adnexal structures - including the conjunctiva, lacrimal gland, and lacrimal drainage system - rather than from the orbit proper [5,6,9,11]. In the present case, involvement of the medial orbit and nasolacrimal duct raises the possibility of origin within the lacrimal drainage apparatus with secondary orbital extension. Among epithelial malignancies of the lacrimal drainage system, nonkeratinizing carcinoma accounts for approximately 15-20% of reported cases [12]. Clinically, lacrimal sac tumors often present with early unilateral epiphora secondary to nasolacrimal duct obstruction [11,13], although this symptom was not observed in this case.

Given the rarity of primary epithelial malignancies arising within the orbit, identification of NKSCC with LE-like features in this location should prompt careful evaluation for secondary involvement. Historical orbital exenteration series have demonstrated that squamous cell carcinoma involving the orbit more commonly originates from adjacent structures including the paranasal sinuses, conjunctiva, skin, and lacrimal drainage system - rather than the orbit proper [14]. In particular, nasopharyngeal carcinoma is well recognized for its potential to extend into or metastasize to the orbit [15]. Consequently, exclusion of an occult nasopharyngeal or sinonasal primary tumor is essential in the diagnostic evaluation of these lesions [16].

Although no clinical or radiologic evidence of a nasopharyngeal or sinonasal lesion was identified in the present case, the absence of dedicated endoscopic evaluation or MRI prevents complete exclusion of a small occult primary lesion.

Regardless of histology, tumors arising primarily within the orbit typically present with progressive but nonspecific symptoms, including proptosis, diplopia, ocular pain or pressure, restricted extraocular movements, and periorbital swelling. Manifestations related to ocular adnexal involvement may occur later in the disease course. Because these findings overlap with those of more common orbital pathologies, delayed diagnosis is not uncommon. At the same time, aggressive lesions may result in visual impairment and substantial functional morbidity.

This case further expands the limited literature on EBV-positive NKSCC involving the orbit and highlights the importance of considering this entity in the differential diagnosis of rapidly progressive orbital masses, particularly when EBV positivity is identified.

## Conclusions

EBV-NKSCC of the orbit is an exceptionally rare malignancy that shares histologic characteristics with nasopharyngeal carcinoma. Its nonspecific clinical presentation may delay diagnosis and treatment. This case highlights NKSCC as an important diagnostic consideration in rapidly progressive orbital masses, particularly in the setting of EBV positivity or Asian ancestry. Early recognition, thorough imaging, and histopathologic confirmation are critical for guiding appropriate management. Reporting such cases helps expand the clinical understanding of this rare entity, highlighting its diagnostic challenges and informing potential therapeutic approaches.
